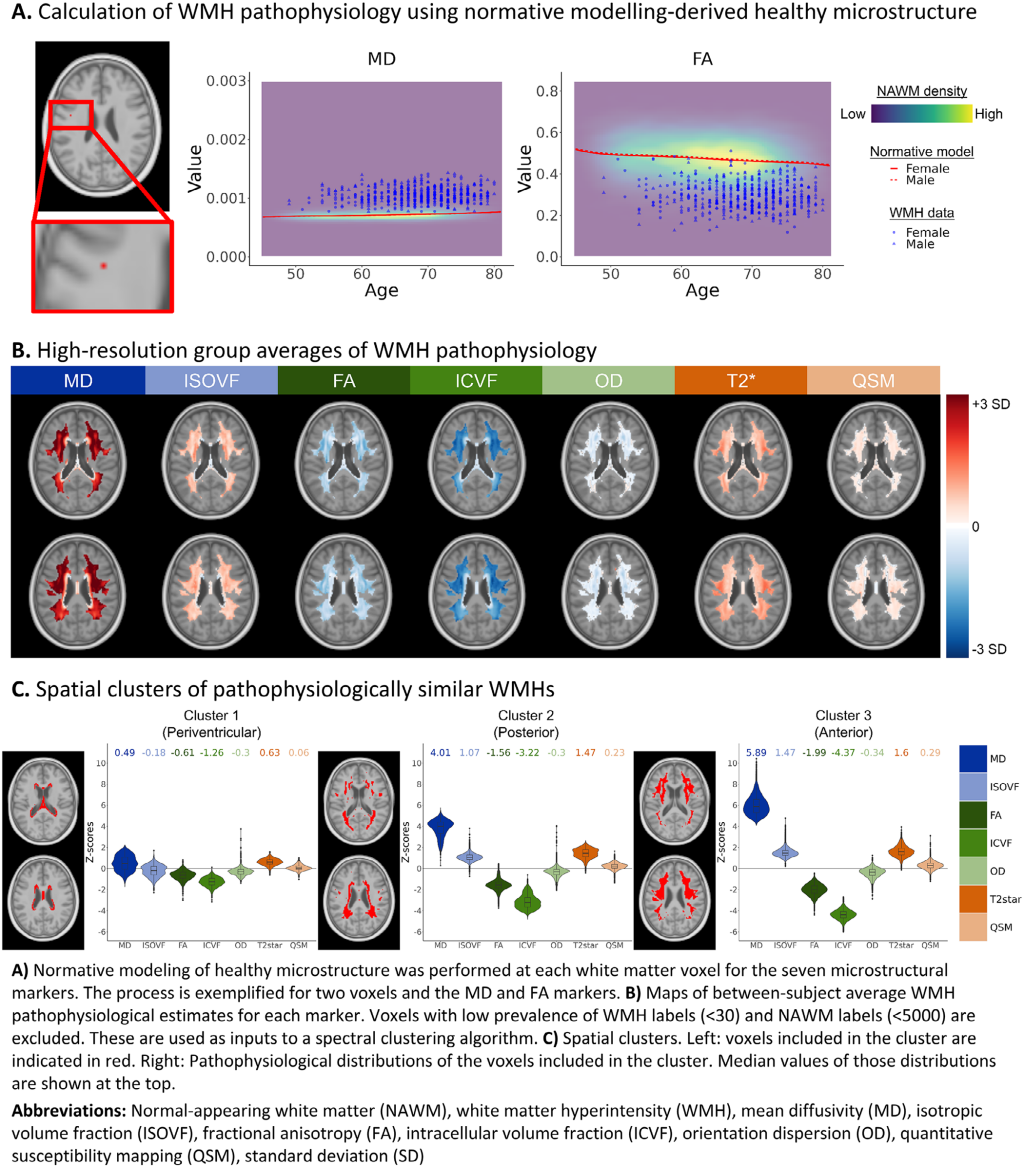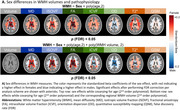# Sex differences in white matter hyperintensity pathophysiology

**DOI:** 10.1002/alz70862_110231

**Published:** 2025-12-23

**Authors:** Olivier Parent, Sophia Osborne, Gabriel A. Devenyi, Aurelie Bussy, Manuela Costantino, Jérémie Fouquet, Daniela Quesada Rodriguez, Mahsa Dadar, Mallar M. Chakravarty

**Affiliations:** ^1^ McGill University, Montreal, QC Canada; ^2^ Douglas Mental Health University Institute, Montreal, QC Canada; ^3^ Douglas Mental Health University Institute, Montréal, QC Canada; ^4^ Department of Psychiatry, McGill University, Montréal, QC Canada; ^5^ Cerebral Imaging Centre, Douglas Mental Health Institute Research Centre, Montreal, QC Canada

## Abstract

**Background:**

White matter hyperintensity (WMH) pathophysiology varies across regions and consists of various degrees of edema, inflammation, demyelination, and axonal degeneration. Notable sex differences in WMH volume have been observed, with women having a higher WMH burden than men starting from midlife, which is hypothesized to be due to the menopausal transition and the consequent reduction in the neuroprotective effects of estrogen. However, a deep characterization of the spatial pathophysiological patterns of WMHs across sexes is lacking.

**Method:**

We estimated WMH pathophysiology *in vivo* at a high spatial resolution using microstructural magnetic resonance imaging (MRI). In the UK Biobank dataset (*n* = 32,526, 15,144 males, 17,382 females), diffusion‐ and susceptibility‐weighted images were used to derive fluid‐, fiber‐, and myelin‐ and iron‐sensitive markers. Age‐ and sex‐specific expected values of healthy white matter microstructure were calculated at a voxel‐level resolution using normative modeling and used to contrast with WMH microstructural values to derive pathophysiological estimates (Figure 1A). We derived spatial clusters of pathophysiologically similar WMHs by applying spectral clustering to group‐level averages of WMH pathophysiology (Figure 1B) resulting in three regions: periventricular, posterior, and anterior (Figure 1C). The median WMH pathophysiology within each region was sampled for each subject. We characterized sex differences in our derived WMH pathophysiological patterns using linear models, controlling for non‐linear age effects and correcting *p*‐values using the false discovery rate.

**Result:**

In general, females showed higher WMH volumes and more severe WMH pathophysiology, with notable exceptions: males showed higher WMH volume and worst orientation dispersion (OD) pathophysiology in posterior WMHs (Figure 2). When investigating WMH pathophysiology for equivalent WMH volume, a clear pattern emerged, with strong effects in females mostly restricted to periventricular and posterior WMHs for most pathophysiological markers. Intriguingly, this was not the case for the OD marker which only showed a significantly higher effect in males in the PV region.

**Conclusion:**

Taken together, our results show nuanced sex‐specific effects in WMHs. There are clear spatial differences, with females having more WMHs but similar pathophysiological effects in anterior WMHs, while males show higher WMH volumes but lower pathophysiological effects in posterior WMHs.